# Supplementary material for: Development of Aptamer-DNAzyme based metal-nucleic acid frameworks for gastric cancer therapy
Source: Nat Commun. 2024 May 1;15:3684. doi: 10.1038/s41467-024-48149-9 (PMC11063048; doi:10.1038/s41467-024-48149-9)
Supplement: Supplementary file 1 — Supplementary Information [file 41467_2024_48149_MOESM1_ESM.docx]

Supporting information

**Development of Aptamer-DNAzyme Based Metal-Nucleic Acid Frameworks for Gastric Cancer therapy**

*Jiaqi Yan^1,2,3^, Rajendra Bhadane^2,4,5^, Meixin Ran^2,3^, Xiaodong Ma^2,3^, Yuanqiang Li^2,3^, Dongdong Zheng^6^, Outi M.H. Salo-Ahen^2,5^, Hongbo Zhang^1,2,3^**

1 Department of Orthopaedics Shanghai Key Laboratory for Prevention and Treatment of Bone and Joint Diseases Shanghai Institute of Traumatology and Orthopaedics Ruijin Hospital Shanghai Jiao Tong University School of Medicine197 Ruijin 2nd Road, Shanghai 200025, P. R. China

2 Pharmaceutical Sciences Laboratory, Faculty of Science and Engineering, Åbo Akademi University, Turku, Finland

3 Turku Bioscience Centre, University of Turku and Åbo Akademi University, Turku, Finland

4 Institute of Biomedicine, University of Turku, Turku, Finland

5 Structural Bioinformatics Laboratory, Biochemistry, Åbo Akademi University, 20520 Turku, Finland

6 Department of Ultrasound, Fudan University Shanghai Cancer Center, Shanghai, 200032, PR China

*Corresponding Author

Hongbo Zhang：hongbo.zhang@abo.fi

**Supplementary Table S1. All sequences used in this work.**

|  | 5' to 3' |
| --- | --- |
| H-GDz for *in vitro* | GCAGCGGTGT GGGGGCAGCG GTGTGGGGGC AGCGGTGTGG GGTTGCACCA GCGAGGCTCT CAGCGAGACG AAATGAGGTG CAT |
| MH-GDz for *in vitro* | **CG**AGCGGTGT GGGG**CG**A**CG**G GTGTGGGG**CG** A**CG**GGTGTGG GGTTGCACCA GCGAGGCTCT CAGCGAGACG AAATGAGGTG CAT |
| H-GDz MB for *in vitro* imaging | GCAGCGGTGT GGGGGCAGCG GTGTGGGGGC AGCGGTGTGG GG/i6FAMdT/TGCACCA GCGAGGCTCT CAGCGAGACG AAATGAGGTG CAT-BHQ1 |
| MH-GDz MB for *in vitro* imaging | **CG**AGCGGTGT GGGG**CG**A**CG**G GTGTGGGG**CG** A**CG**GGTGTGG GG/i6FAMdT/TGCACCA GCGAGGCTCT CAGCGAGACG AAATGAGGTG CAT-BHQ1 |
| H-GDz for *lysosome escape* | GCAGCGGTGT GGGGGCAGCG GTGTGGGGGC AGCGGTGTGG GGTTGCACCA GCGAGGCTCT CAGCGAGACG AAATGAGGTG CAT-Cy5.5 |
| H-MGDz for in vitro | GCAGCGGTGT GGGGGCAGCG GTGTGGGGGC AGCGGTGTGG GGTTGCACCA GCGAGGCTCT C**CGA**GAGA**GC** AAATGAGGTG CAT |
| GDz for in vitro | TTGCACCAGC GAGGCTCTCA GCGAGACGAA ATGAGGTGCAT |
| GLUT-1 Substrate for MNF synthesis | TGCACCTCATAGGCCTCGCT |
| GLUT-1 mRNA Substrate for *in vitro* cleavage | UGCACCUCAUAGGCCUCGCU |
| H-GDz MB for *in vivo* imaging | GCAGCGGTGT GGGGGCAGCG GTGTGGGGGC AGCGGTGTGG GG/iCY5.5dT/TGCACCA GCGAGGCTCT CAGCGAGACG AAATGAGGTG CAT-BHQ3 |
| MH-GDz MB for *in vivo* imaging | **CG**AGCGGTGT GGGG**CG**A**CG**G GTGTGGGG**CG** A**CG**GGTGTGG GG/iCY5.5dT/TGCACCA GCGAGGCTCT CAGCGAGACG AAATGAGGTG CAT-BHQ3 |
| H-GDz for *in vivo* | GCAGCGGTGT GGGGGCAGCG GTGTGGGGGC AGCGGTGTGG GGTTGCACCA GCGAGGCTCT CAGCGAGACG AAATGAGGTG CAT |
| H-MGDz for *in vivo* | GCAGCGGTGT GGGGGCAGCG GTGTGGGGGC AGCGGTGTGG GGTTGCACCA GCGAGGCTCT C**CGA**GAGA**GC** AAATGAGGTG CAT |

**Supplementary Tables S2. Structural composition of the MNF.**

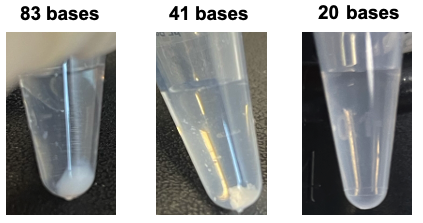


**Supplementary Figure 1.** Photographs of DNA fragments with the same number of nucleotides but different lengths incubated with calcium ions and subjected to centrifugation. (n = 3 independent experiments with similar results).

**Short strand GDz**


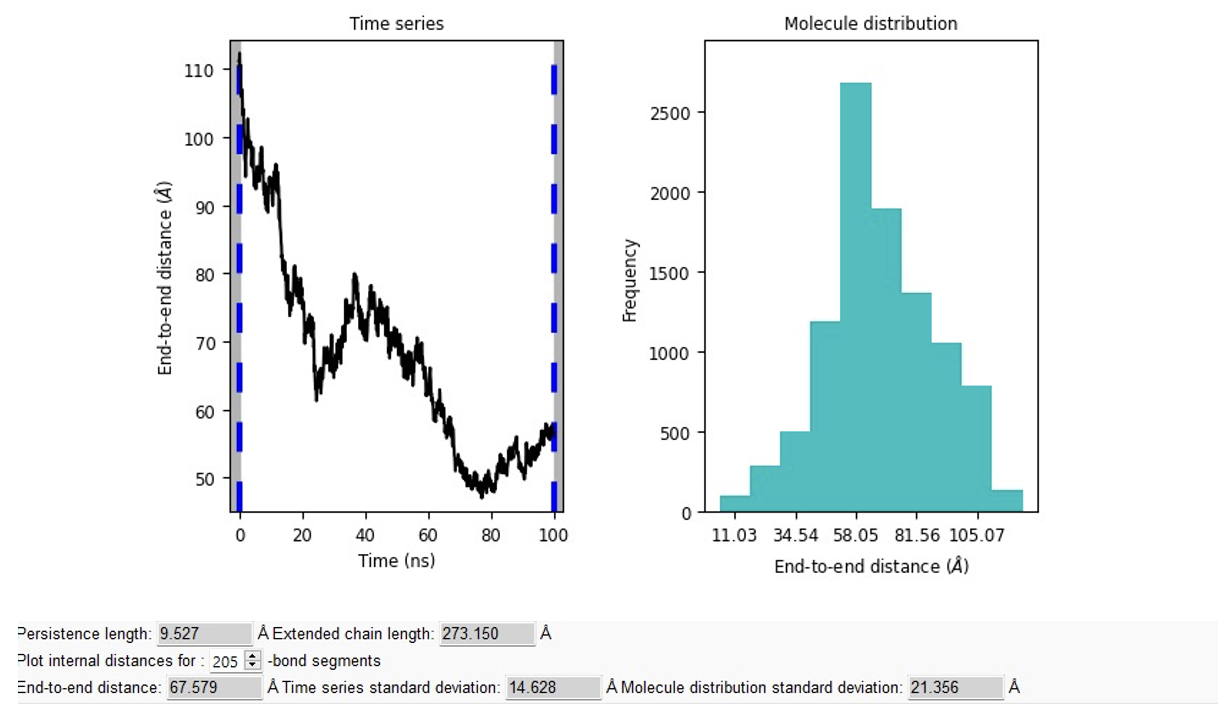


**long strand H-GDz**


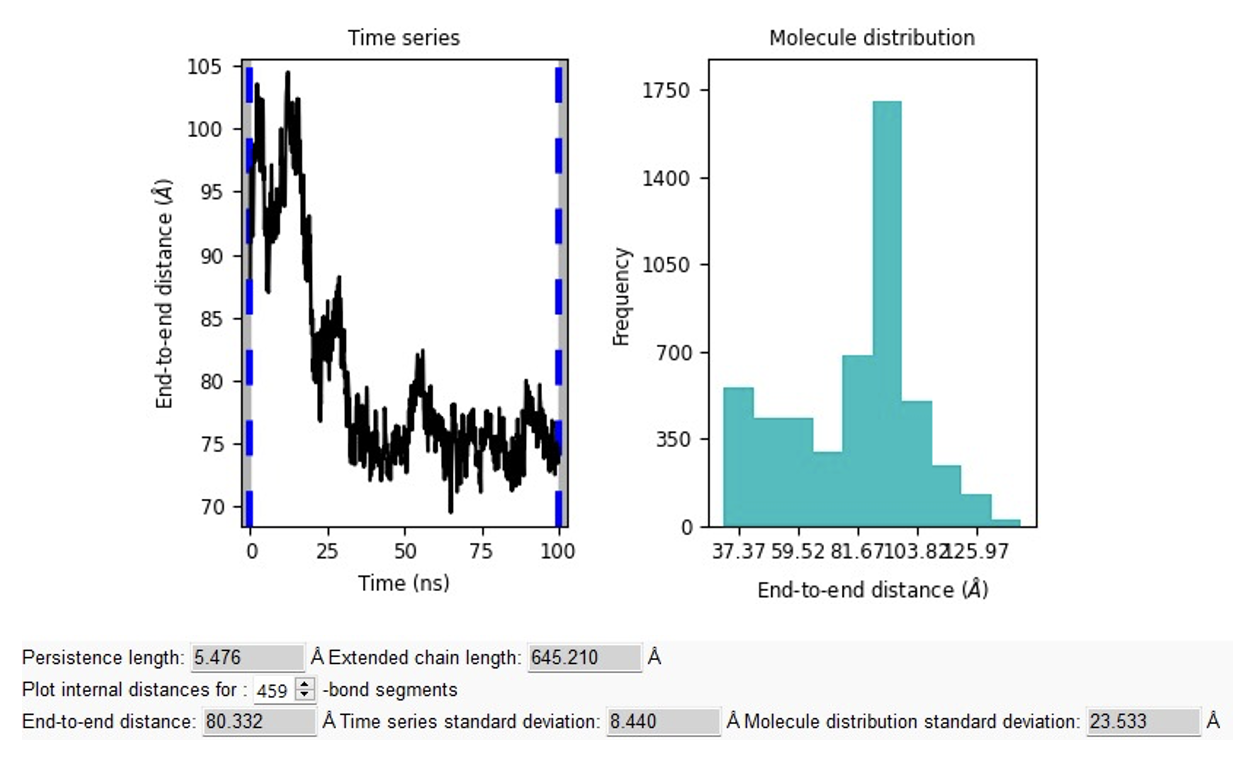


**Supplementary Figure 2.** The persistence length of short strand GDz, and the persistence length of long strand H-GDz. The source data is available in the provided Source Data file.

**Supplementary Figure 3.** Conformational changes of a DNA sequence fragment containing 83 nucleotides in a solution containing only sodium chloride. The results indicate that sodium ions do not promote DNA aggregation and MNF material formation.


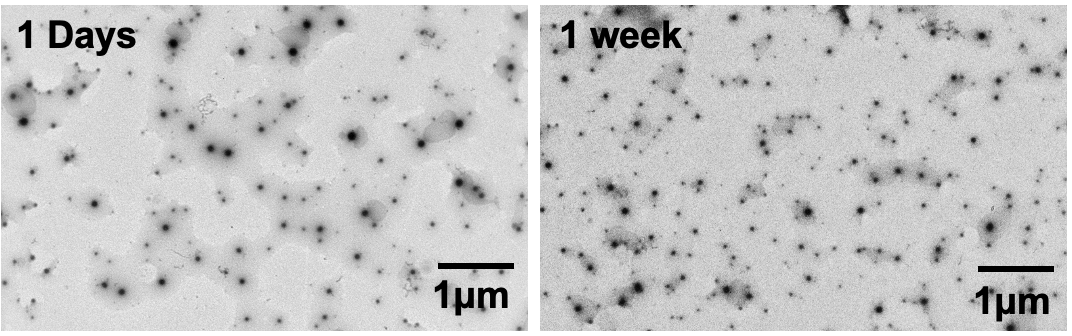


**Supplementary Figure 4.** The TEM image of MNF material after storage in DMEM for one week. (n = 3 independent experiments with similar results).


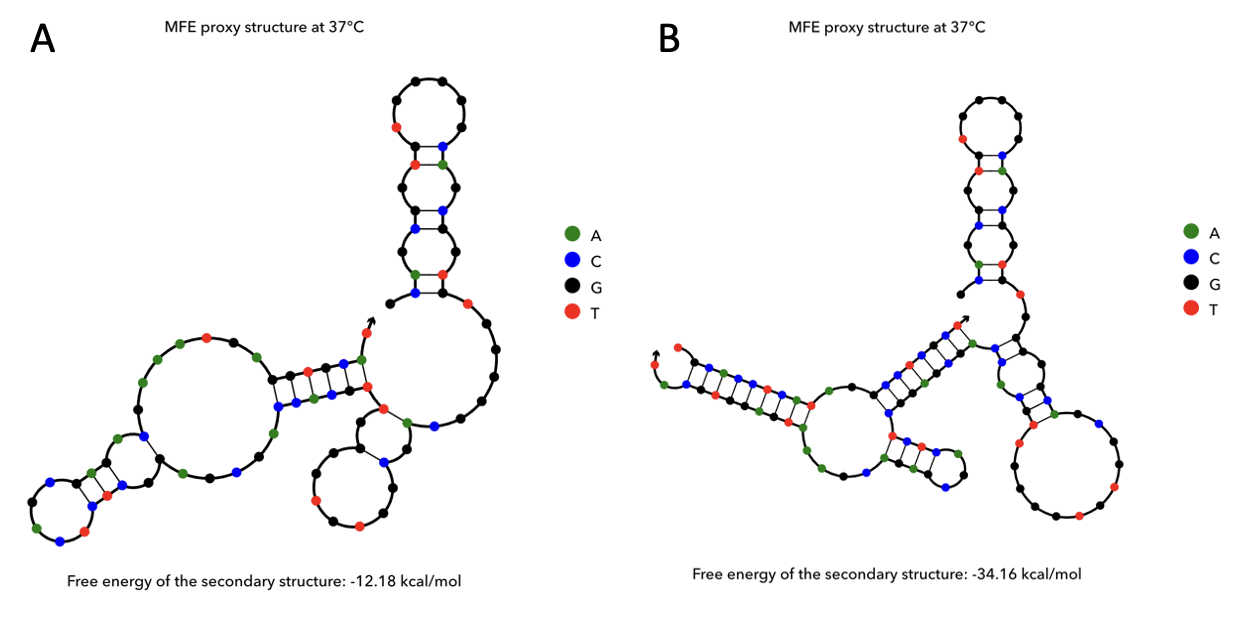


**Supplementary Figure 5.** Binding of H-GDz sequence to GLUT-1 mRNA substrate. (A) Secondary structure of the H-GDz sequence. (B) Complex structure formed by the binding of H-GDz to GLUT-1 mRNA substrate. The experimental results were simulated using the NUPACK Web App^1,2^.

**Supplementary Figure 6.** Densitometric analysis of the bands through ImageJ. (n = 3 independent experiments and the data are presented as mean values ± SD) All statistics were calculated using one-way ANOVA using a Tukey post hoc test. Source data are provided as a Source Data file.


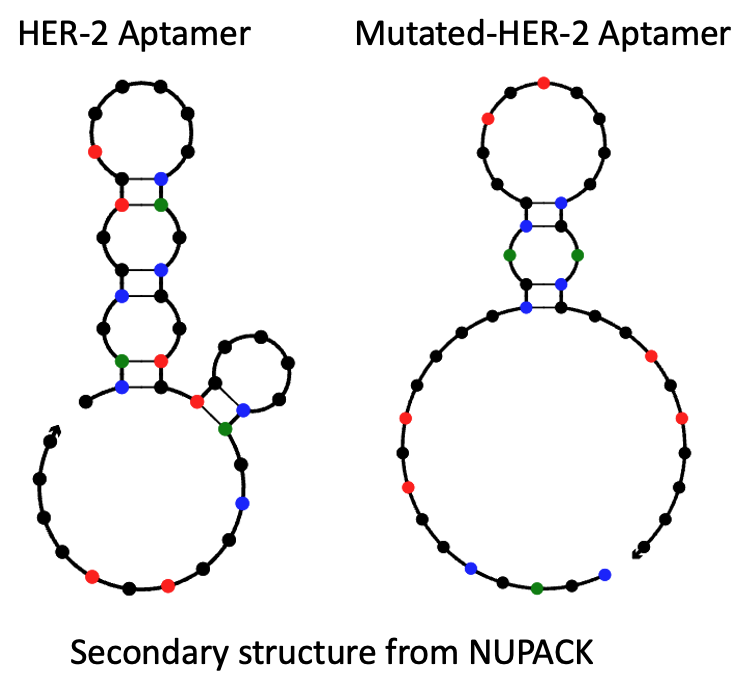


**Supplementary Figure 7.** Simulated the secondary structures of HER-2 aptamer before and after mutation using the NUPACK software.


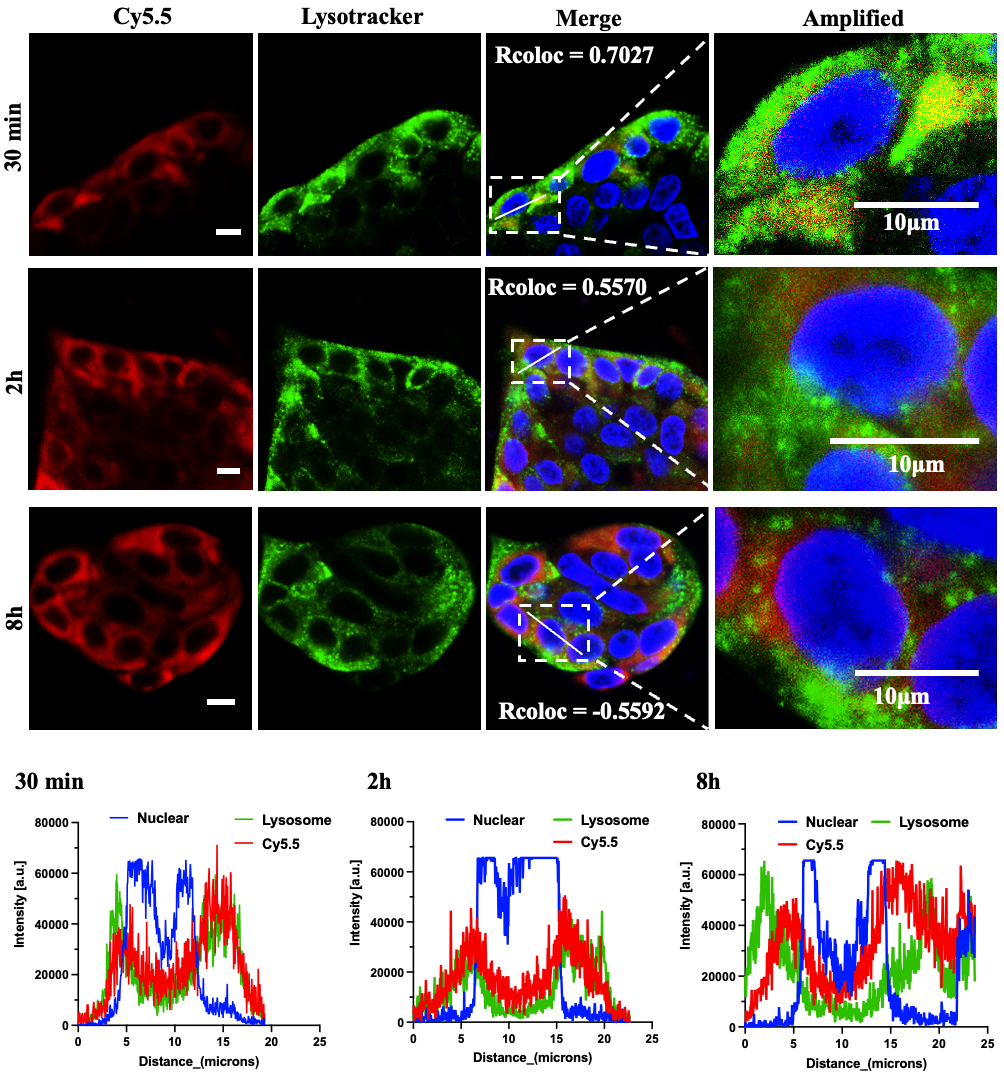


**Supplementary Figure 8.** Lysosomal escape experiment of MNF material. The MNF material was synthesized using Cy5.5-labeled H-GDz, allowing the observation of the intracellular localization of MNF material through the Cy5.5 channel (red signal). Lysosomes within the cells were detected using the green channel of LysoTracker™ Green DND-26. The co-localization analysis of the two channels within the cells was performed using the Coloc 2 plugin and line-scan analysis in ImageJ. (n = 3 independent experiments, with similar results. Source data are provided as a Source Data file.)


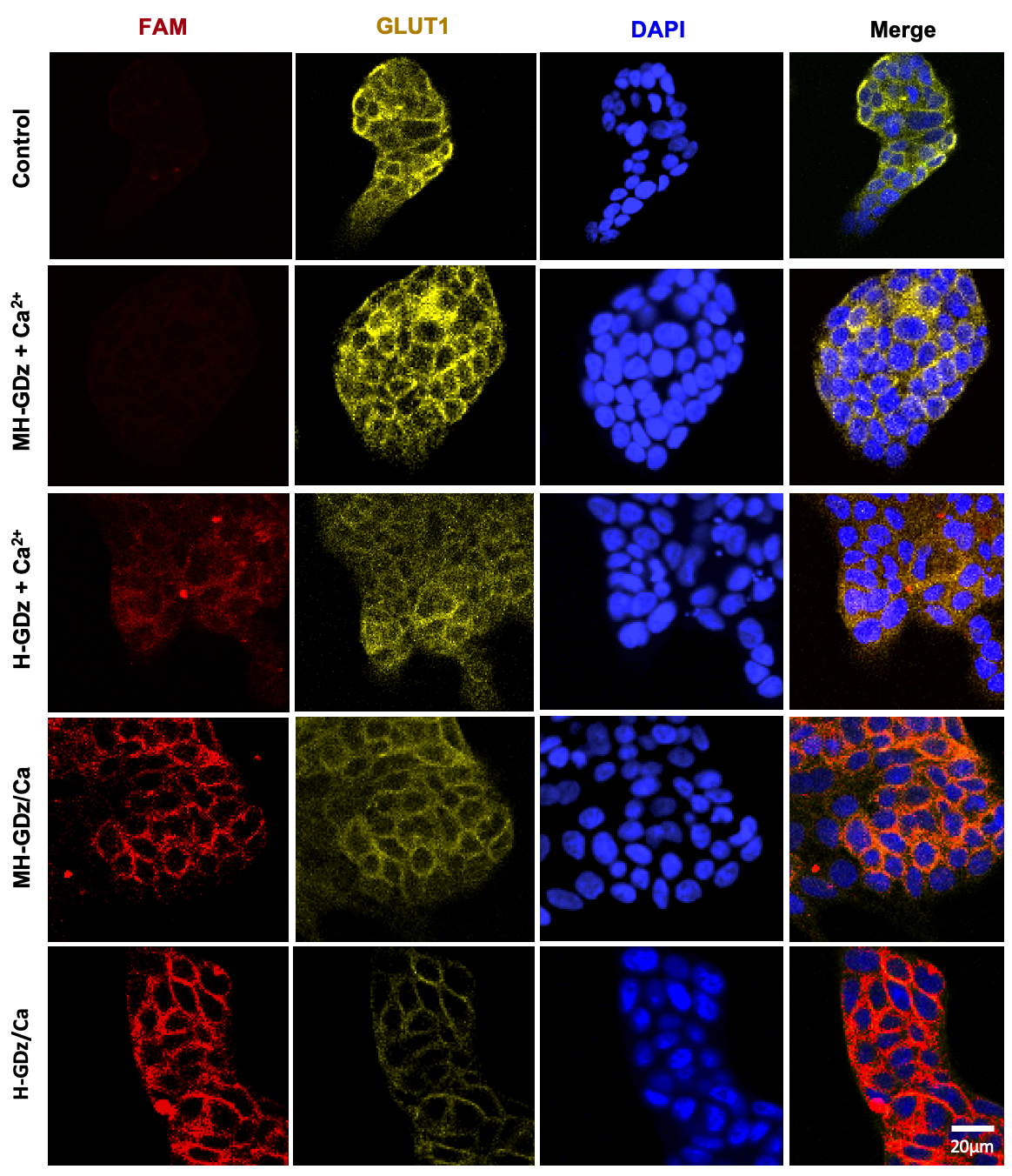


**Supplementary Figure 9.** Higher magnification to facilitate a clear observation of intracellular protein expression and fluorescence signals. (n = 3 independent experiments, with similar results.)

A B


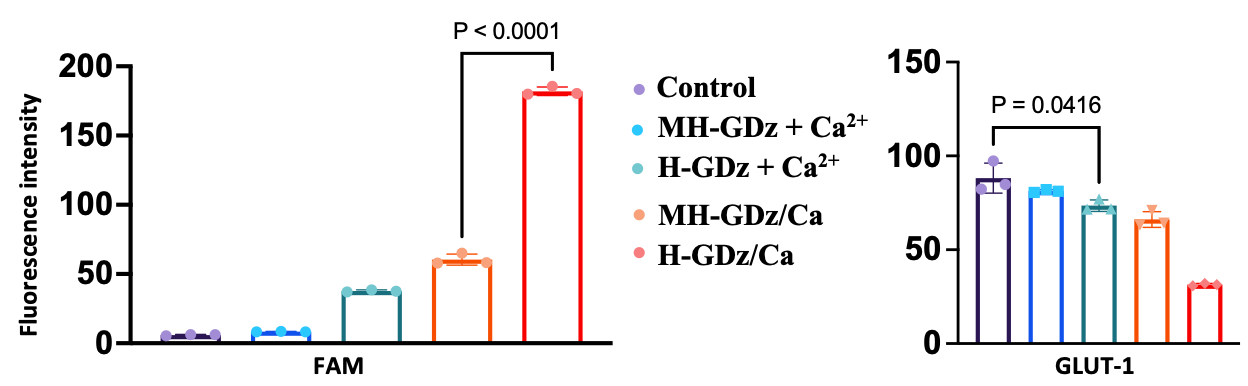


**Supplementary Figure 10.** Quantification of fluorescence signal intensity in confocal microscopy experiments using ImageJ. (A) Quantification of fluorescence intensity emitted in the FAM channel (470/525 nm) after binding of GDz to mRNA. (B) Immunofluorescence experiment targeting GLUT-1, labeled in the TRICT channel (557/576 nm). (n = 3 independent experiments and the data are presented as mean values ± SD) Statistics were calculated using two-tailed paired t-test for the two interested group. Source data are provided as a Source Data file.


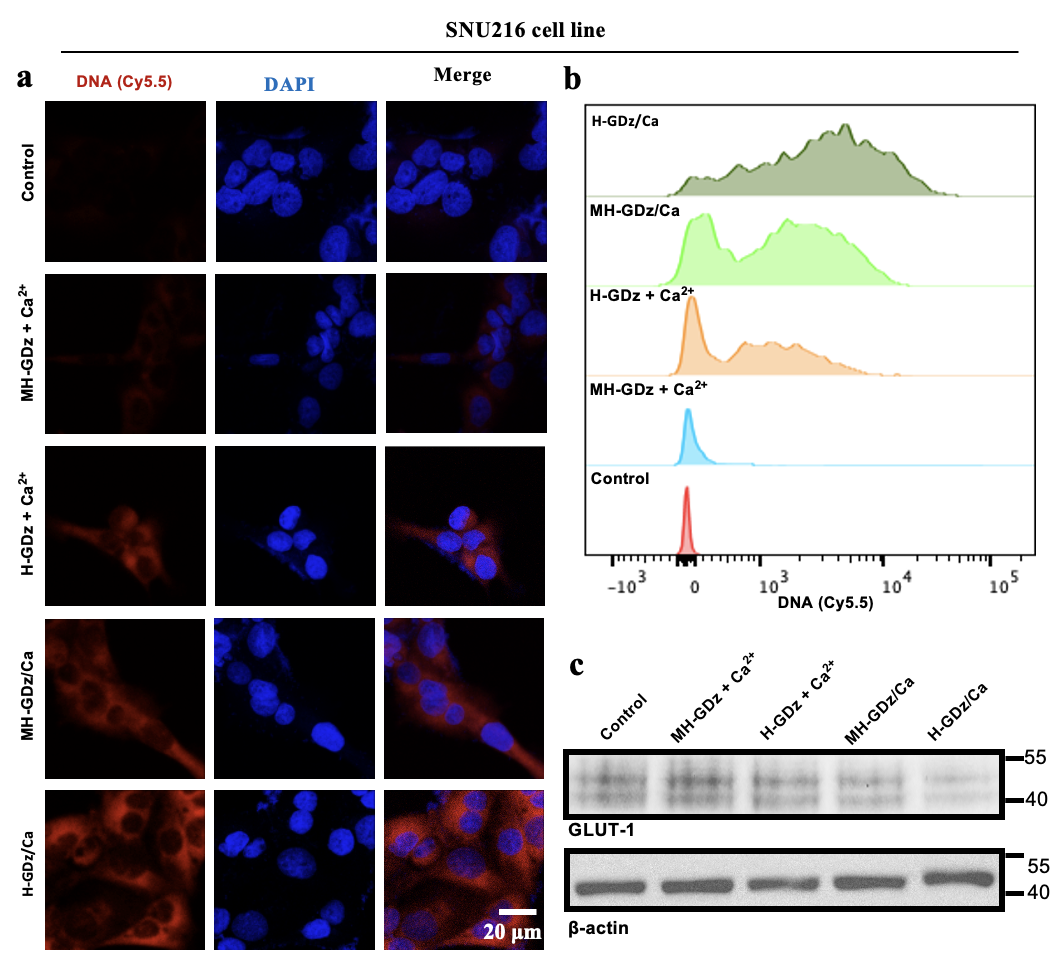


**Supplementary Figure 11.** The experiments with MNF materials on SNU216 cells. The H-GDz sequence was labeled with Cy5.5 and shown as red signal, while the cell nuclear was labeled by DAPI (Blue color) (a) Confocal experiments to confirm the cellular uptake of each material. (b) Flow cytometry techniques to verify the internalization of nanomaterials by SNU216 cells. (c) Assessment of the regulation of GLUT-1 within cells for each material. (n = 3 independent experiments with similar results)


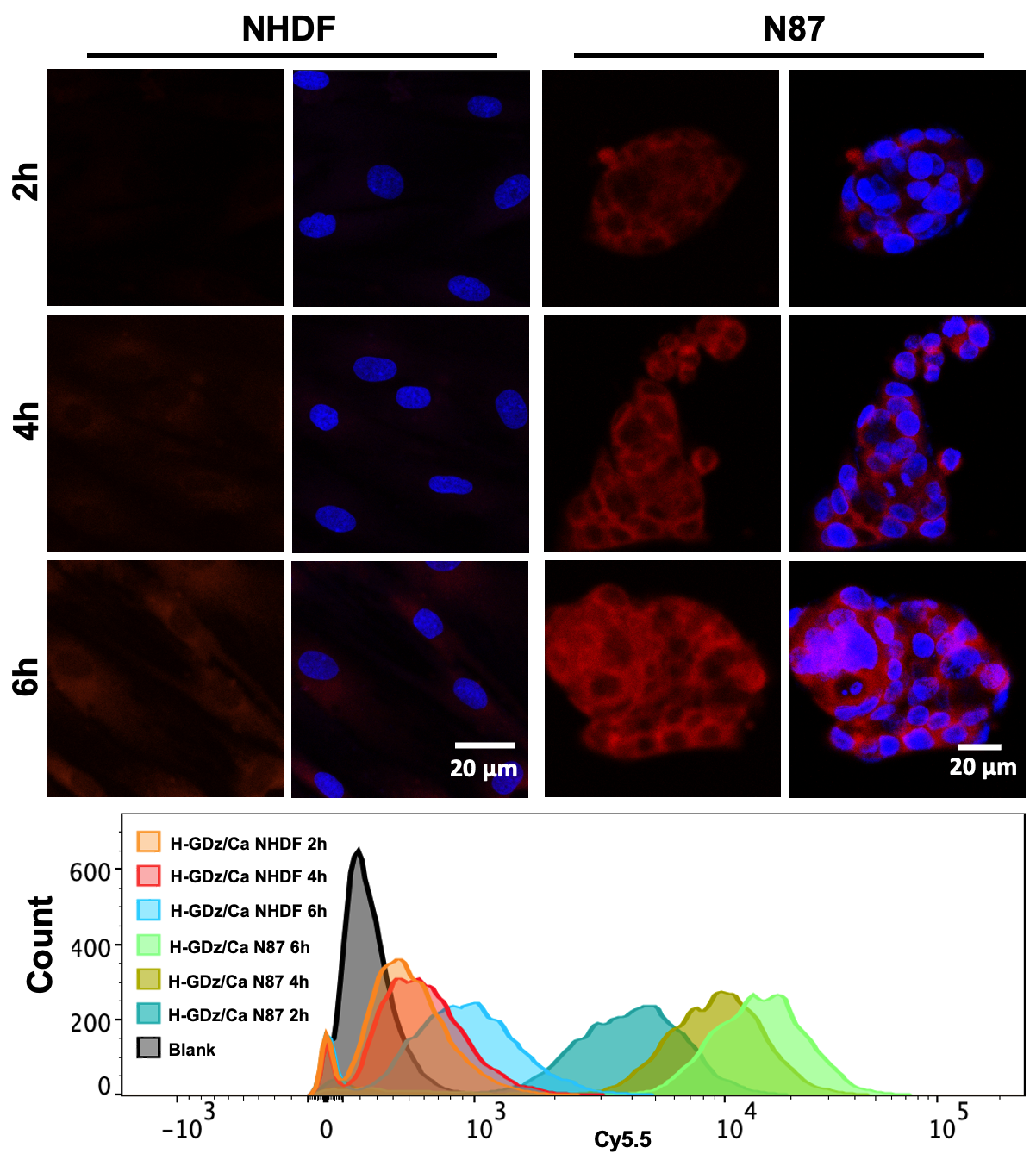


**Supplementary Figure 12.** The internalization experiments of MNF materials on different HER-2 expressing cell lines involved confocal microscopy and flow cytometry. Confocal experiments and flow cytometry techniques were employed to validate the uptake of H-GDz/Ca MNF NPs by both human dermal fibroblast cells (NHDF) and gastric cancer N87 cells. The H-GDz sequence was labeled with Cy5.5 and shown as red signal, while the cell nuclear was labeled by DAPI (Blue color). (n = 3 independent experiments with similar results.)


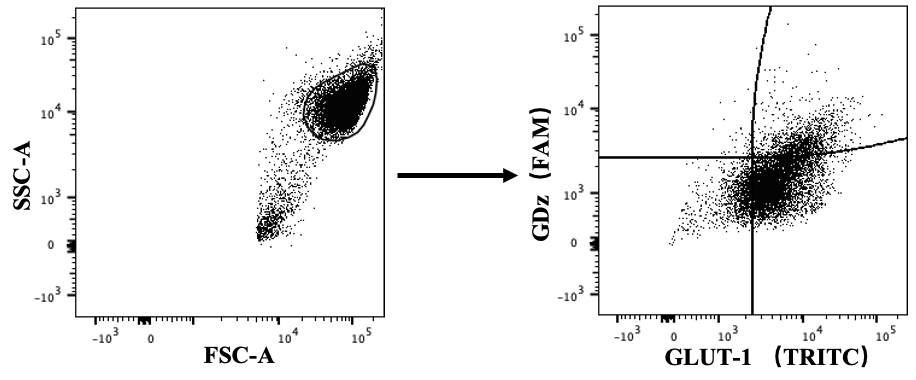


**Supplementary Figure 13.** Gating strategy for analyzing the silencing efficacy of different groups on GLUT-1 expression. (Account for Figure 2f)


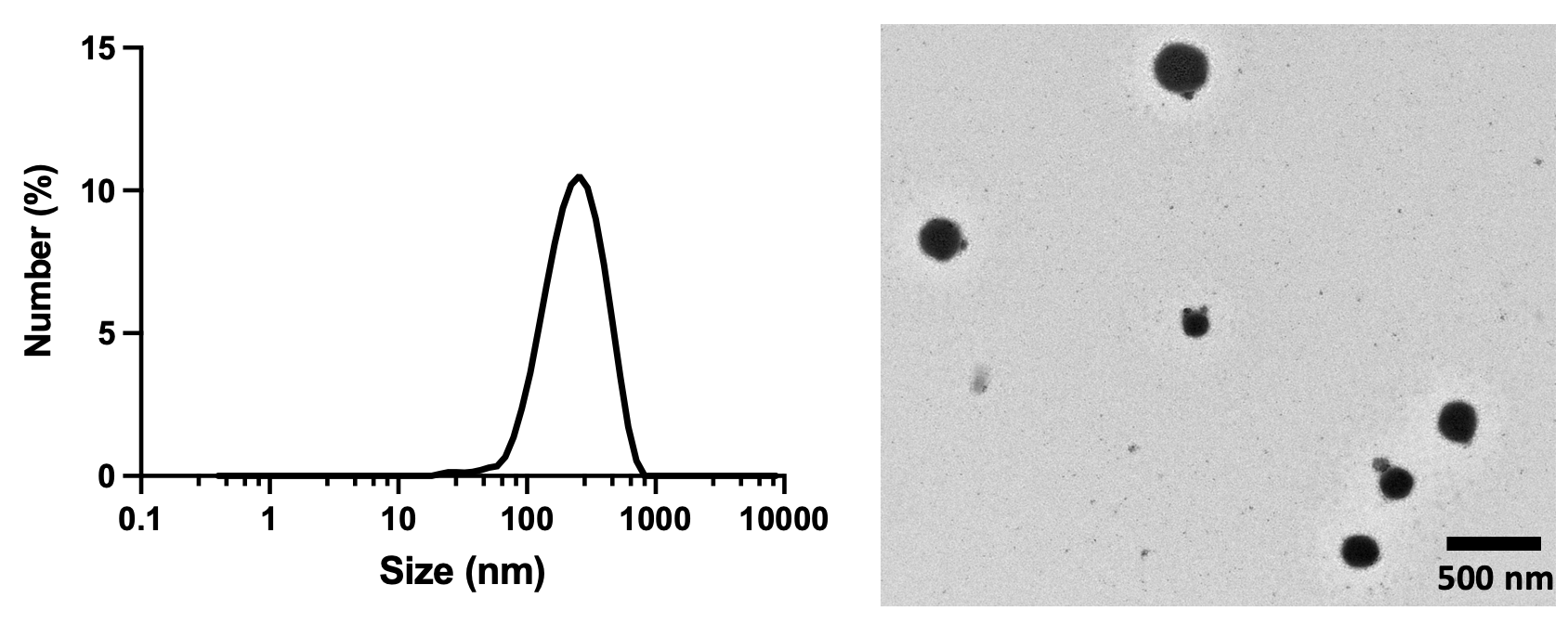


**Supplementary Figure 14.** Characterization of H-GDz/Ca MNF materials after loading IRF-1 protein. (n = 3 independent experiments with similar results, Source data are provided as a Source Data file.)


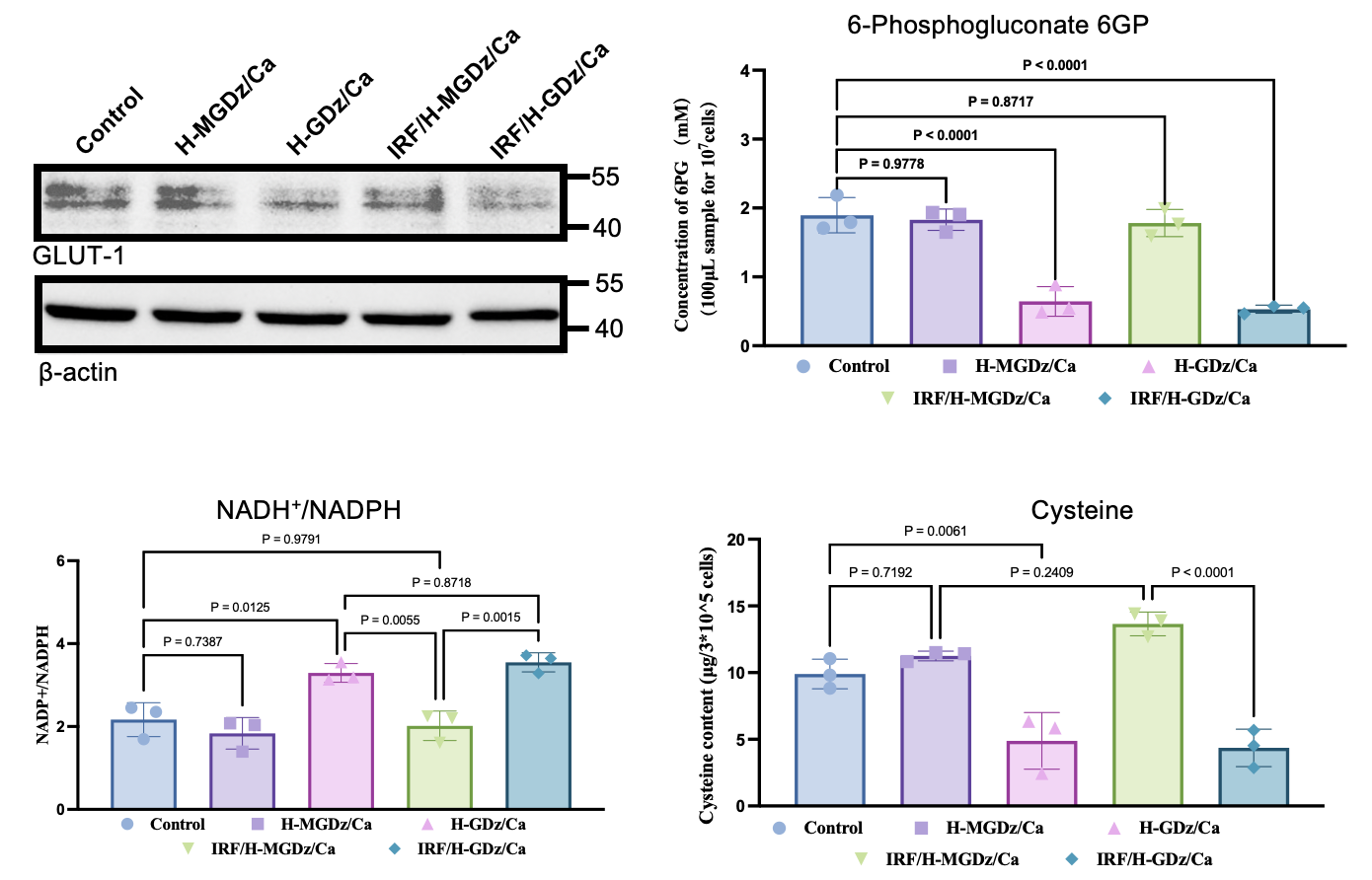


**Supplementary Figure 15.** Regulation of metabolic pathways in N87 cells by different MNF materials. Characterization of GLUT-1 protein expression in N87 cells by different material groups, along with downstream characterization of PPP pathway and hexose phosphorylation pathways. (n = 3 independent experiments and the data are presented as mean values ± SD) All statistics were calculated using one-way ANOVA using a Tukey post hoc test. Source data are provided as a Source Data file.


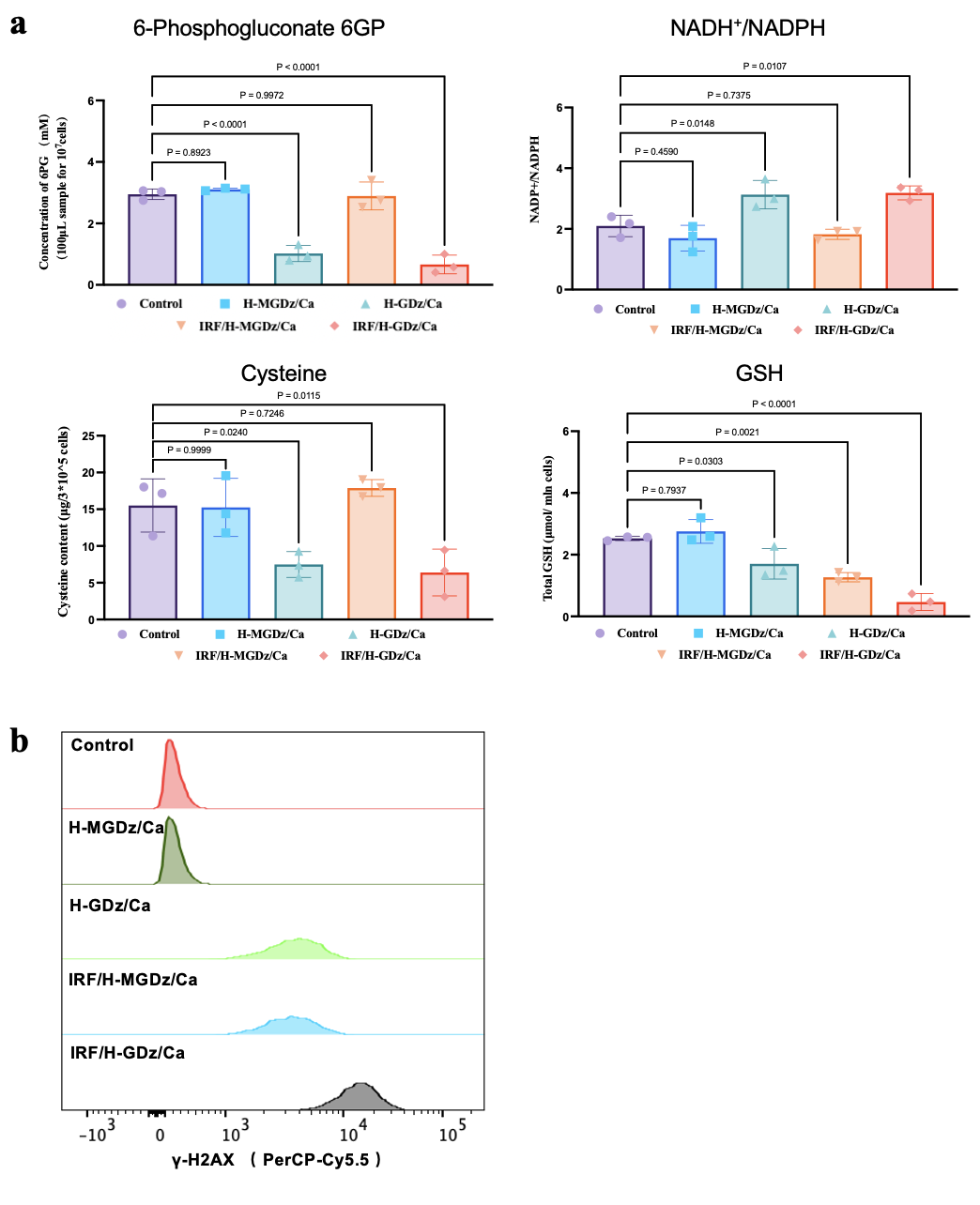


**Supplementary Figure 16.** Regulation of metabolic pathways in SNU216 cells by different MNF materials. (a) Characterization of intracellular PPP metabolism, NADH, and Cysteine consumption processes, as well as GSH synthesis in SNU216 cells. (b) Analysis of the nuclear damage marker γ-H2AX using flow cytometry. (n = 3 independent experiments and the data are presented as mean values ± SD) All statistics were calculated using one-way ANOVA using a Tukey post hoc test. Source data are provided as a Source Data file.

**Supplementary Figure 17.** Observation of nuclear damage in cells under different treatment conditions using TEM. In the control and H-MGDz/Ca groups, the nuclei appear intact with evenly distributed internal DNA. In the H-GDz/Ca group, there are few black aggregates within the DNA, indicating DNA damage and condensation. In the IRF/H-MGDz/Ca group, nuclear morphology is distorted, and numerous black aggregates are present, indicating nuclear damage. In the IRF/H-GDz/Ca group, severe nuclear condensation is observed, with extensive DNA damage within the nuclei. (n = 3 independent experiments, with similar results.)

**Supplementary Figure 18.** Pharmacokinetics of IRF/H-GDz/Ca and IRF/MH-GDz/Ca NPs through blood circulation. (n = 3 independent experiments and the data are presented as mean values ± SD) Source data are provided as a Source Data file.

**
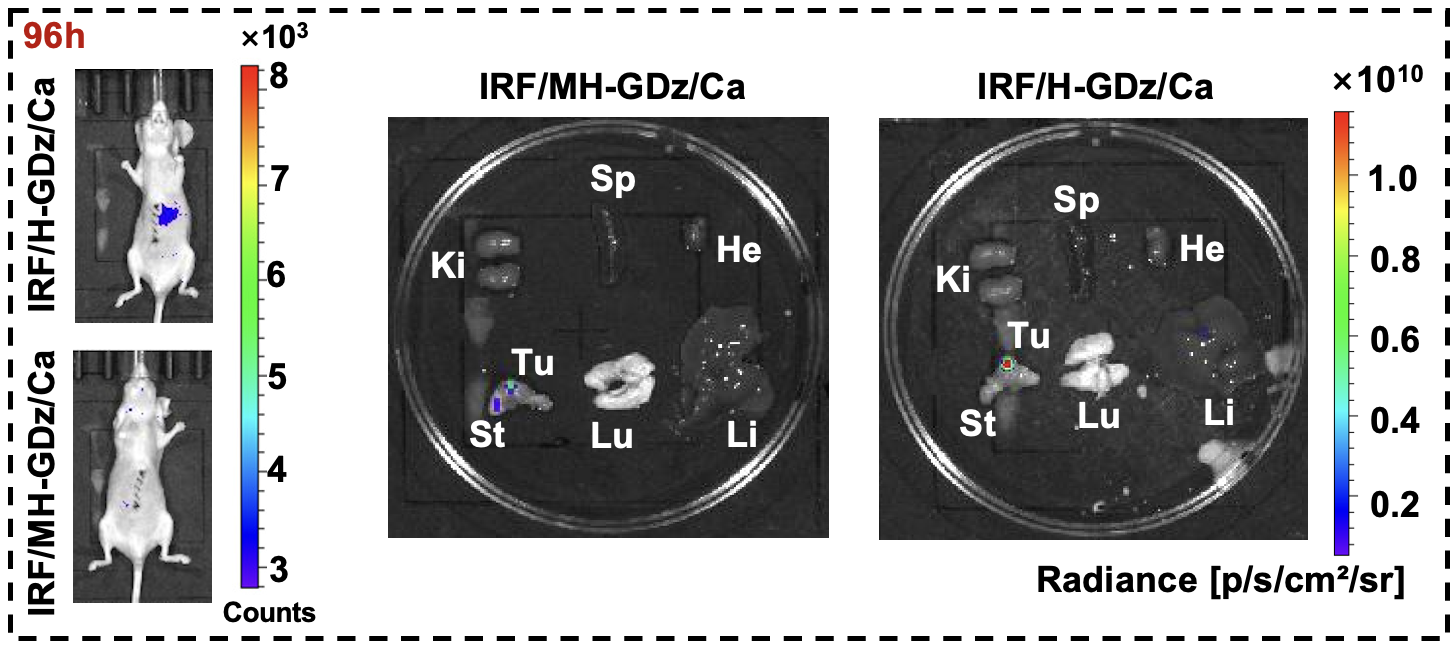
**

**Supplementary Figure 19.** Intravenous administration of MNF materials and their distribution in mice at 96h timepoint. (n = 3 independent experiments with similar results).


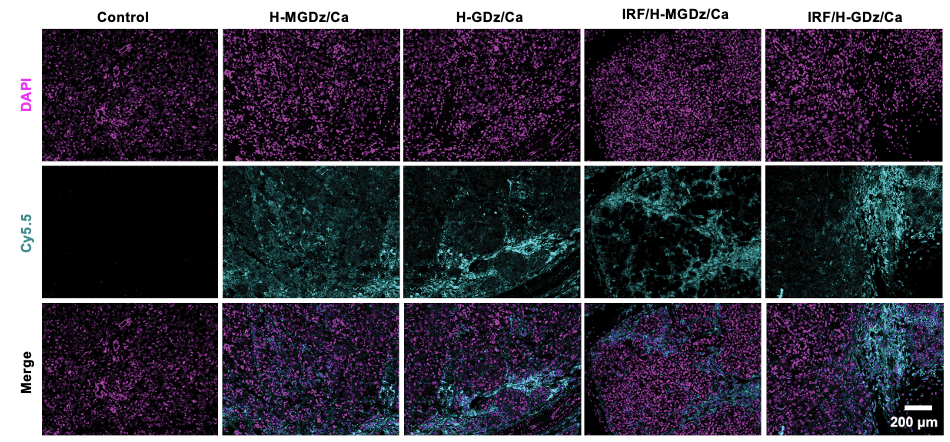


**Supplementary Figure 20.** Imaging of the materials in the tumor site of mice, DAPI staining was employed to label the cell nuclei. (n = 4 independent experiments with similar results).

**Supplementary Figure 21**. Ex vivo photographs of gastric organ with gastric tumor.

**
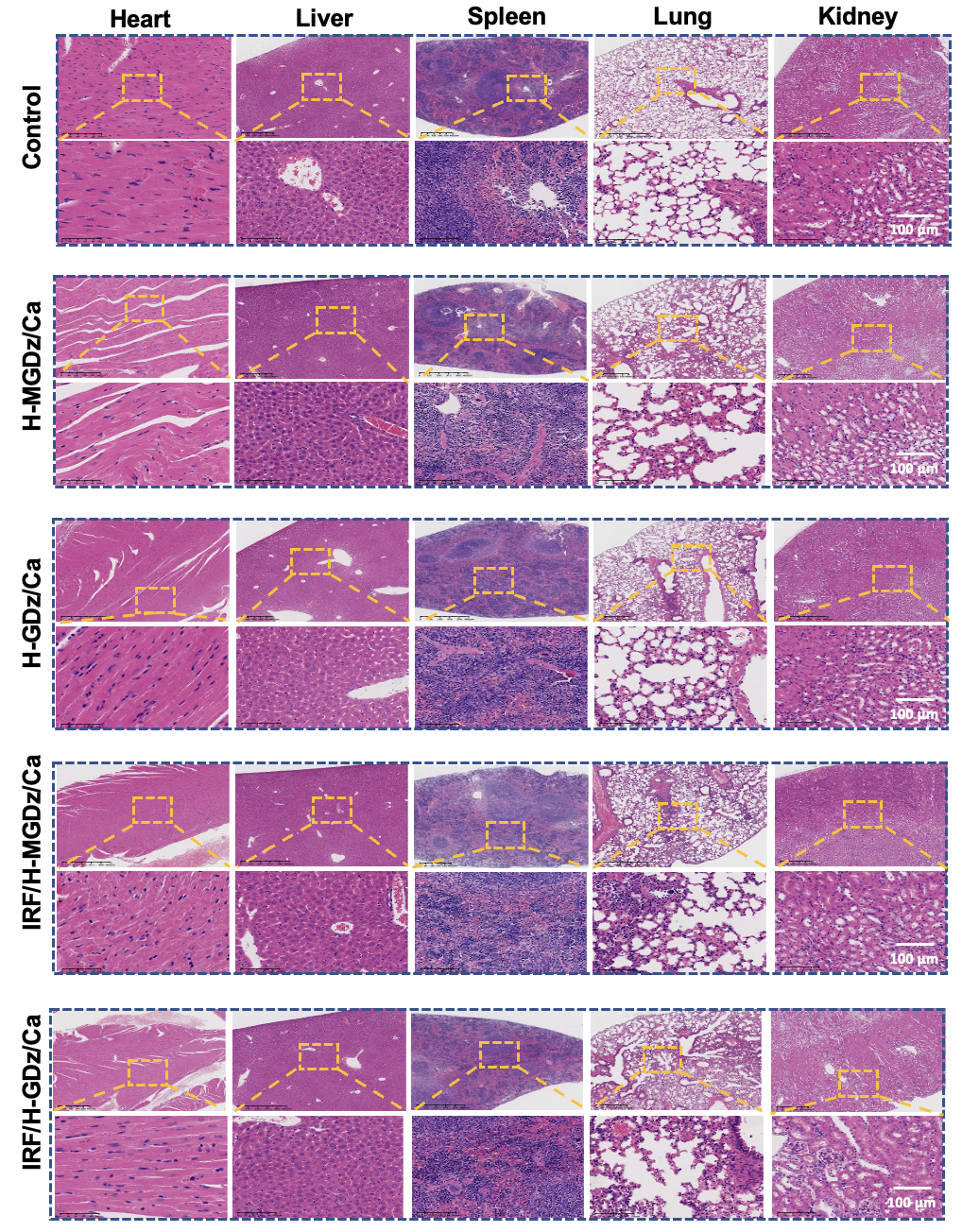
**

**Supplementary Figure 22.** H&E stained tissue sections of heart, liver, spleen, lung and kidney. (n = 4 independent experiments with similar results).


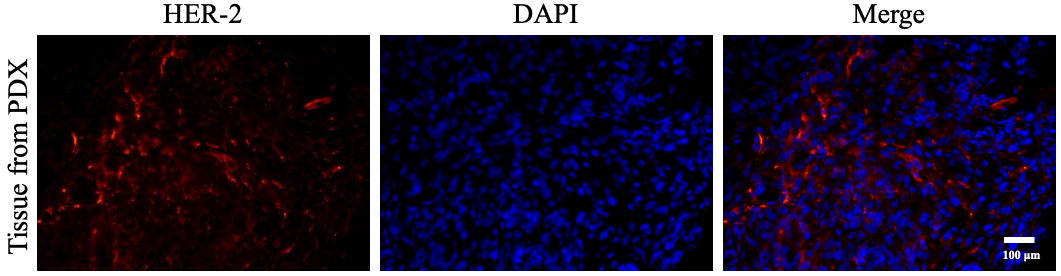


**Supplementary Figure 23.** The tissue sections from the constructed PDX model were used for HER-2 staining. (n = 4 independent experiments with similar results).

**Supplementary Figure 24.** Tumor growth and weight for different groups for the PDX model. (n = 4 independent experiments and the data are presented as mean values ± SD) Source data are provided as a Source Data file.


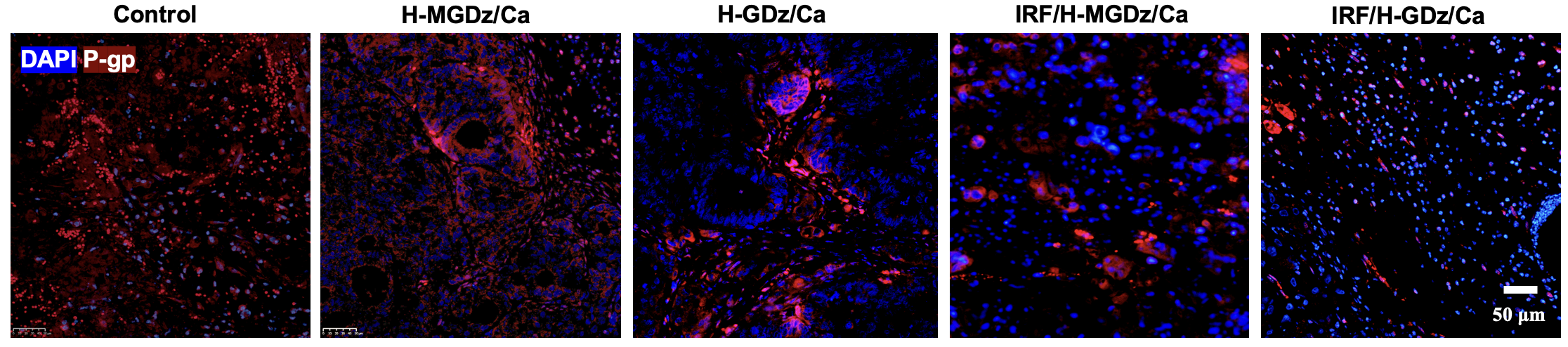


**Supplementary Figure 25.** Immunofluorescence detection of P-glycoprotein (P-gp) for the PDX model. (n = 4 independent experiments with similar results.)


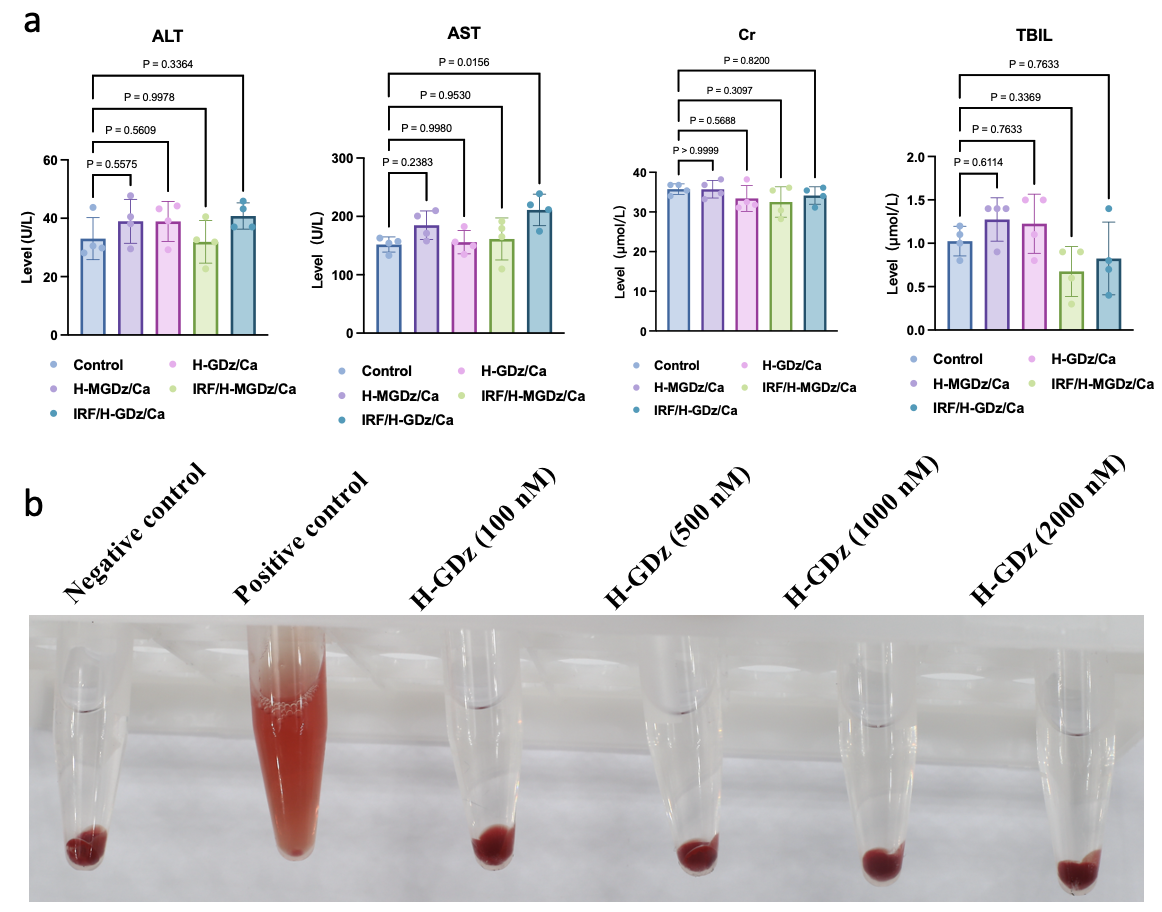


**Supplementary Figure 26.** The blood biochemistry and Hemolysis test. (a) After intravenous injection of different NPs, the blood biochemistry of mice was measured, with untreated mice as the control. (b) Hemolysis test conducted for the IRF/H-GDz/Ca group. ALT: Alanine Aminotransferase; AST: Aspartate Aminotransferase; Cr: Creatinine, TBIL: Total Bilirubin. (n = 4 independent experiments and the data are presented as mean values ± SD) All statistics were calculated using one-way ANOVA using a Tukey post hoc test. Source data are provided as a Source Data file.

References

1. Fornace ME, Huang J, Newman CT, Porubsky NJ, Pierce MB, Pierce NA. NUPACK: Analysis and Design of Nucleic Acid Structures, Devices, and Systems. ChemRxiv. Cambridge: Cambridge Open Engage; 2022; This content is a preprint and has not been peer-reviewed.
2. Zadeh, J.N., Steenberg, C.D., Bois, J.S., Wolfe, B.R., Pierce, M.B., Khan, A.R., Dirks, R.M. and Pierce, N.A. (2011), NUPACK: Analysis and design of nucleic acid systems. J. Comput. Chem., 32: 170-173. https://doi.org/10.1002/jcc.21596
