# Supplementary material for: Development of Aptamer-DNAzyme based metal-nucleic acid frameworks for gastric cancer therapy
Source: Nat Commun. 2024 May 1;15:3684. doi: 10.1038/s41467-024-48149-9 (PMC11063048; doi:10.1038/s41467-024-48149-9)
Supplement: Supplementary file 2 — Reporting Summary [file 41467_2024_48149_MOESM2_ESM.pdf]

nature portfolio

Corresponding author(s): Hongbo Zhang

Last updated by author(s): Mar 30, 2024

Reporting Summary

Nature Portfolio wishes to improve the reproducibility of the work that we publish. This form provides structure for consistency and transparency in reporting. For further information on Nature Portfolio policies, see our Editorial Policies and the Editorial Policy Checklist.

Statistics

For all statistical analyses, confirm that the following items are present in the figure legend, table legend, main text, or Methods section.

n/a	Confirmed	
☐	☒	The exact sample size (n) for each experimental group/condition, given as a discrete number and unit of measurement
☐	☒	A statement on whether measurements were taken from distinct samples or whether the same sample was measured repeatedly
☐	☒	The statistical test(s) used AND whether they are one- or two-sided<br>*Only common tests should be described solely by name; describe more complex techniques in the Methods section.*
☒	☐	A description of all covariates tested
☐	☒	A description of any assumptions or corrections, such as tests of normality and adjustment for multiple comparisons
☐	☒	A full description of the statistical parameters including central tendency (e.g. means) or other basic estimates (e.g. regression coefficient) AND variation (e.g. standard deviation) or associated estimates of uncertainty (e.g. confidence intervals)
☐	☒	For null hypothesis testing, the test statistic (e.g. F, t, r) with confidence intervals, effect sizes, degrees of freedom and P value noted<br>*Give P values as exact values whenever suitable.*
☒	☐	For Bayesian analysis, information on the choice of priors and Markov chain Monte Carlo settings
☒	☐	For hierarchical and complex designs, identification of the appropriate level for tests and full reporting of outcomes
☒	☐	Estimates of effect sizes (e.g. Cohen's d, Pearson's r), indicating how they were calculated

Our web collection on statistics for biologists contains articles on many of the points above.

Software and code

Policy information about availability of computer code

Data collection	Transmission electron microscope (TEM) : JEM-1400 PLUS TEM; Microplate reader: SkanIt 2.4.3.37; Maestro Molecular Modeling Suite : Schrödinger Release 2023-1, New York, USA; DNA weight : NanoDrop™ 2000/2000c Spectrophotometers, Thermo Scientific™; Particle size investigation: Zetasizer Nano ZS instrument; Scanning Electron Microscope (SEM) : Thermo Scientific Apreo S field-emission scanning electron microscope; Energy Dispersive Spectrometer (EDS) : Oxford Instruments UltimMax 100 energy dispersive X-ray spectrometer; Fluorescence Imaging System : ChemiDoc MP imaging system (Bio-Rad); Confocal laser scanning microscopy: Zeiss LSM 880 with AiryScan ZEN 2.3 SP1 black edition; Flow Cytometer: BD LSR Fortessa analyzers; Gel Imaging System : GelDoc Go Gel Imaging System (Bio-RAD); IVIS® Spectrum In Vivo Imaging System:IVIS Lumina Series III
Data analysis	ImageJ; Flowjo_V10; GraphPad prism 9.0

For manuscripts utilizing custom algorithms or software that are central to the research but not yet described in published literature, software must be made available to editors and reviewers. We strongly encourage code deposition in a community repository (e.g. GitHub). See the Nature Portfolio guidelines for submitting code & software for further information.

Data

Policy information about availability of data

All manuscripts must include a data availability statement. This statement should provide the following information, where applicable:

- Accession codes, unique identifiers, or web links for publicly available datasets
- A description of any restrictions on data availability
- For clinical datasets or third party data, please ensure that the statement adheres to our policy

Data availability
All data supporting the findings of this study are available within the Article, Supplementary Information, or the Source Data file. The source data generated during this study have been deposited in the Figshare database, https://doi.org/10.6084/m9.figshare.25041767. The source data are provided along with this paper.

Research involving human participants, their data, or biological material

Policy information about studies with human participants or human data. See also policy information about sex, gender (identity/presentation), and sexual orientation and race, ethnicity and racism.

Reporting on sex and gender	Samples used were from adults no prior knowledge of sex and gender
Reporting on race, ethnicity, or other socially relevant groupings	Samples were from adults with no prior knowledge of race or ethnicity
Population characteristics	The current study only involved obtaining excised tissue from a human carcinoma patient after surgery, with informed consent.
Recruitment	A gastric cancer patient who underwent surgical treatment at Fudan University Shanghai Cancer Center. There was no potential self-selection bias.
Ethics oversight	Approval for human experiment was granted by the Ethics Committee of Fudan University Shanghai Cancer Center (Approval No.:050432-4-2108*). Written and informed consent was procured from patient before the collection of tissue samples. All experimental procedures adhered to ethical standards and international guidelines, including the "International Ethical Guidelines for Biomedical Research Involving Human subjects" "Declaration of Helsinki".

Note that full information on the approval of the study protocol must also be provided in the manuscript.

Field-specific reporting

Please select the one below that is the best fit for your research. If you are not sure, read the appropriate sections before making your selection.

[x] Life sciences [ ] Behavioural & social sciences [ ] Ecological, evolutionary & environmental sciences

For a reference copy of the document with all sections, see nature.com/documents/nr-reporting-summary-flat.pdf

Life sciences study design

All studies must disclose on these points even when the disclosure is negative.

Sample size	No statistical method was used to predetermine sample size.<br><br>For in vitro experiments, we opted for 3 independent replications to strike a balance between cost-effectiveness and obtaining a distribution closer to the true one.<br><br>For in vivo antitumor studies , each group included 4 individuals to assess statistical significance, to strike a balance between cost-effectiveness and obtaining a distribution closer to the true one. The specific 'n' values for each figure are provided in the figure legends. Due to the low variability observed between samples, we considered this to be sufficient.
Data exclusions	No data was excluded from studies.
Replication	We confirmed that all repeated attempts were successful. Experiment repeat numbers are reported in Figure Legends.
Randomization	Samples were randomly allocated into experimental groups.
Blinding	The investigators were blinded to group allocation during data collection and analysis.

Reporting for specific materials, systems and methods

We require information from authors about some types of materials, experimental systems and methods used in many studies. Here, indicate whether each material, system or method listed is relevant to your study. If you are not sure if a list item applies to your research, read the appropriate section before selecting a response.

Materials & experimental systems

n/a	Involved in the study
	x Antibodies
	x Eukaryotic cell lines
x	Palaeontology and archaeology
	x Animals and other organisms
x	Clinical data
x	Dual use research of concern
x	Plants

Methods

n/a	Involved in the study
x	ChIP-seq
	x Flow cytometry
x	MRI-based neuroimaging

Antibodies

Antibodies used

The following antibodies were used for western blot. They are listed as antigen first, followed by supplier, catalog number
1) Anti-Rabbit Glut-1 antibody, Abcam, ab115730, 1: 100000 dilution;
2) Anti-Rabbit RAD51 antibody, Abcam, ab133534, 1: 1000 dilution;
3) Anti-Mouse β-Actin antibody, Abcam, ab8226, 1: 2000 dilution;
4) Anti-Rabbit FAP antibody, Abcam, ab314456, 1: 1000 dilution;
5) Anti-Rabbit alpha smooth muscle Actin antibody, Abcam, ab5694, 1: 1000 dilution;
6) Anti-Rabbit PGP antibody, Abcam, ab261736, 1: 1000 dilution;
7) Anti-Rabbit gamma H2A.X (phospho S139) antibody, Abcam, ab81299, 1: 1000 dilution;
8) Anti-Rabbit Alexa Fluor® 488 Anti-HMGB1 antibody, Abcam, ab195010, 1: 1000 dilution

Validation

The species and application of the following antibodies used for western blot were validated by the manufacturer.
1) Anti-Rabbit Glut-1 antibody, https://www.abcam.com/en-fi/search?sorting=relevance&keywords=glut-1
2) Anti-Rabbit RAD51 antibody,https://www.abcam.com/en-fi/search?sorting=relevance&keywords=RAD51
3) Anti-Mouse β-Actin, https://www.abcam.com/en-fi/search?sorting=relevance&keywords=beta-Actin
4) Anti-Rabbit FAP antibody, https://www.abcam.com/en-fi/search?sorting=relevance&keywords=FAP
5) Anti-Rabbit alpha smooth muscle Actin antibody, https://www.abcam.com/en-fi/search?sorting=relevance&keywords=alpha%20smooth%20muscle%20Actin
6) Anti-Rabbit PGP antibody, https://www.abcam.com/en-fi/search?sorting=relevance&keywords=PGP
7) Anti-Rabbit gamma H2A.X (phospho S139) antibody, https://www.abcam.com/en-fi/search?sorting=relevance&keywords=gamma%20H2A.X%20(phospho%20S139)
8) Anti-Rabbit Alexa Fluor® 488 Anti-HMGB1 antibody, https://www.abcam.com/en-fi/search?sorting=relevance&keywords=Alexa%20Fluor%C2%AE%20488%20Anti-HMGB1

Eukaryotic cell lines

Policy information about cell lines and Sex and Gender in Research

Cell line source(s)

Human gastric cancer NCI-N87 cells were purchased from ATCC®, normal human dermal fibroblasts NHDF cells were purchased from PromoCell®, human gastric cancer SNU-216 cells were purchased from shanghai biowing applied biotechnology.

Authentication

The N87 and NFDH cell lines used were directly bought from ATCC® and PromoCell®, without any authentication. The SNU-216 cells were authenticated through Short tandem repeat (STR) testing.In detail, DNA extraction will be performed using the Axygen Genomic DNA Extraction Kit. Subsequently, DNA samples will undergo amplification using the 21-STR amplification scheme, followed by the detection of the STR loci and the gender gene Amelogenin on the ABI3730XL genetic analyzer. Appropriate positive and negative controls were run and confirmed for the sample.

Mycoplasma contamination

The cell line tested negative for mycoplasma contamination.

Commonly misidentified lines
(See ICLAC register)

No commonly misidentified cell lines were used in the study.

Animals and other research organisms

Policy information about studies involving animals; ARRIVE guidelines recommended for reporting animal research, and Sex and Gender in Research

Laboratory animals

All PDX experiments were conducted in 6-8-week-old female NOG (NOD.Cg-Prkdcscid IL2rgtm1Sug/JicCrl) mice provided by Beijing Vital River Laboratory Animal Technology Co., Ltd.
BALBc-nu(6-8 weeks old) were supplied by the Animal Center of Hangzhou Medical College. The living environment of animals were maintained at a temperature of ~25 °C with a 12 h light/dark cycle, humidity at 50-60%, with free access to standard food and water.

Wild animals	The study did not involve wild animals.
Reporting on sex	To prevent mice from the same group from mating and producing offspring, only one sex (female) was chosen. However, we believe that the research results were applicable to all sex.
Field-collected samples	The study did not involve samples collected from the field.
Ethics oversight	Ethical statement<br>Our study strictly followed ethical guidelines, with all animal procedures conducted in accordance with the Guidelines for the Care and Use of Laboratory Animals. Approval was obtained from the Institutional Animal Care and Use Committee (IACUC) of Zhejiang Center of Laboratory Animals (ZJCLA), under the reference number ZJCLA-IACUC-20010304. All experimental procedures adhered to ethical standards and international guidelines, including the "International Ethical Guidelines for Biomedical Research Involving Human subjects" "Declaration of Helsinki".

Note that full information on the approval of the study protocol must also be provided in the manuscript.

Plants

Seed stocks	*Report on the source of all seed stocks or other plant material used. If applicable, state the seed stock centre and catalogue number. If plant specimens were collected from the field, describe the collection location, date and sampling procedures.*
Novel plant genotypes	*Describe the methods by which all novel plant genotypes were produced. This includes those generated by transgenic approaches, gene editing, chemical/radiation-based mutagenesis and hybridization. For transgenic lines, describe the transformation method, the number of independent lines analyzed and the generation upon which experiments were performed. For gene-edited lines, describe the editor used, the endogenous sequence targeted for editing, the targeting guide RNA sequence (if applicable) and how the editor was applied.*
Authentication	*Describe any authentication procedures for each seed stock used or novel genotype generated. Describe any experiments used to assess the effect of a mutation and, where applicable, how potential secondary effects (e.g. second site T-DNA insertions, mosiacism, off-target gene editing) were examined.*

Flow Cytometry

Plots

Confirm that:

☒ The axis labels state the marker and fluorochrome used (e.g. CD4-FITC).

☒ The axis scales are clearly visible. Include numbers along axes only for bottom left plot of group (a 'group' is an analysis of identical markers).

☒ All plots are contour plots with outliers or pseudocolor plots.

☒ A numerical value for number of cells or percentage (with statistics) is provided.

Methodology

Sample preparation	The cells were fixed and pipetted evenly.
Instrument	BD LSR Fortessa analyzers
Software	Flowjo_V10
Cell population abundance	No sorting was performed.
Gating strategy	Generally, cells were first gated on FSC/SSC.

☒ Tick this box to confirm that a figure exemplifying the gating strategy is provided in the Supplementary Information.
